# Chimeric Autoantibody Receptor- and/or Peptide-MHC-Based CAR Therapies for Targeted Elimination of Antigen-Specific B or T Cells in Hypersensitivity Disorders Such as Allergies and Autoimmune Diseases

**DOI:** 10.3390/cells14100753

**Published:** 2025-05-21

**Authors:** Isidora Protić-Rosić, Al Nasar Ahmed Sehgal, Sebastian Wrighton, Birgit Heller, Winfried F. Pickl

**Affiliations:** 1 Center for Pathophysiology, Infectiology and Immunology, Institute of Immunology, Medical University of Vienna, 1090 Vienna, Austria; isidora.protic-rosic@meduniwien.ac.at (I.P.-R.); al.sehgal@meduniwien.ac.at (A.N.A.S.); sebastian.wrighton@gmail.com (S.W.); 2 Faculty of Chemistry, University of Belgrade, 11000 Belgrade, Serbia; 3 University Library, Medical University of Vienna, 1090 Vienna, Austria; birgit.heller@meduniwien.ac.at; 4 Karl Landsteiner University of Health Sciences, 3500 Krems, Austria

**Keywords:** chimeric antigen receptor, CAR, chimeric autoantigen receptor, CAAR, hypersensitivity, allergy, autoimmunity

## Abstract

Hypersensitivity reactions are dysregulated and potentially devastating immune responses, characterized by a tendency to become chronic. They target either self-proteins or harmless foreign proteins and are driven by both T and B cells. Although numerous symptomatic treatment options for hypersensitivity reactions have been established over recent decades, only a few antigen-specific, causal approaches capable of specifically targeting the pathogenic autoreactive T and/or B cells have been developed. Among these are cell-based treatment modalities involving chimeric antigen receptor (CAR)- or chimeric autoantibody-receptor (CAAR)-expressing cells. These therapies utilize B- or T-cell antigens, presented as B-cell epitopes or peptide-major histocompatibility complexes (pMHCs) to serve as bait. The latter are coupled to potent activation domains derived from the TCR/CD3 complex itself, such as the zeta or CD3 chains, as well as domains from bona fide co-stimulatory molecules (e.g., CD28, 4-1BB). Recent in vitro and in vivo studies have demonstrated the therapeutic potential of these ATMP-based strategies in eliminating autoreactive lymphocytes and alleviating hypersensitivity reactions. This systematic review provides a comprehensive overview of the current status of antigen-specific CAR and CAAR T-cell therapies, highlighting novel directions as well as the ongoing challenges within this promising research field.

## 1. Introduction

Biologics such as monoclonal antibodies and advanced therapy medicinal products (ATMPs) have revolutionized the way we treat cancers, autoimmune diseases, and allergies. These therapies have even shown potential to cure diseases in a subset of treated patients; however, they often lack specificity for the targeted cellular population or factor (e.g., cytokine). While the first cancer therapies based on antibodies were clonotype-specific [1], subsequent developments shifted away from specificity toward malignant cells, instead targeting entire cellular populations or substantial subsets thereof [2,3]. Similar developments were observed in the field of ATMP development, especially when we take a closer look at CAR T cells. In this context, the initially developed CAR T cells, which exhibited broadly generic cell-type specificity, were subsequently followed by the creation of clonotype-specific CAR T cells. Interestingly, however, these clonotype-specific CAR T cells have not yet become established therapies. Instead, CAR T cells with a very broad target specificity were developed for clinical use, ultimately leading to the clinically approved CAR therapies targeting CD19 and B-cell maturation antigen (BCMA) [4,5].

While modern medicine increasingly emphasizes individualized patient- and disease-specific approaches (personalized medicine) [6], the providers of clinical CAR T-cell therapies have predominantly focused on CAR T cells targeting differentiation antigens rather than clonotypic lymphocyte receptors. This is despite the apparent feasibility of developing and employing clonotype-specific CAR T cells, which would likely reduce the toxicity of these ATMPs, provide exceptional specificity, and avoid the disruption or eradication of entire cell lineages essential for patient health. The aim of this literature review is to compile past and current knowledge on the generation and targeting of antigen-specific CAR T cells, specifically focusing on chimeric autoantibody receptors (CAARs) and pMHC-based CARs, to evaluate their potential for clinical applications in hypersensitivity disorders such as allergies and autoimmune diseases.

The end of the 20th century was marked by significant advances in cell-based therapies [7]. Beginning with the first successful application of adoptive cell transfer (ACT) in the 1980s and continuing through the development of gene transfer techniques in the 1990s, the advancement and clinical implementation of chimeric antigen receptor (CAR) T-cell therapy for malignancies has revolutionized cancer immunotherapy, providing unprecedented clinical outcomes [8]. Thirty years after the creation of the first-generation CAR T cells, history was made on 30 August 2017, when the U.S. Food and Drug Administration (FDA) approved the first CAR-based gene therapy—anti-CD19 CAR T cells—for the treatment of pediatric and young-adult patients with B-cell acute lymphoblastic leukemia [4,9]. This breakthrough prompted extensive efforts aimed at expanding this therapeutic approach, not only within the field of immune-oncology but also for the treatment of other disorders, including various forms of immune-mediated hypersensitivity reactions. The basic principle of CAR therapy lies in redirecting cytotoxic lymphocytes—most commonly T cells but sometimes also NK cells—to target specific antigens expressed on malignant cells via engineered extracellular binding domains. CARs are fusion proteins composed of extracellular binding domains specific for the target antigen, linked to a transmembrane domain and one or more intracellular signaling domains [10]. Considerable efforts have been made to optimize the number and combination of intracellular signaling domains to enhance both the memory function and cytotoxicity of the resulting CAR-bearing cells. The selective use and strategic arrangement of signaling domains have led to the development of novel CAR generations characterized by reduced tonic signaling activity yet enhanced in vivo memory function and cytotoxicity [11]. Another facet of CAR-based optimization has focused on developing “off-the-shelf”, readily available therapies which would eliminate the need for patient-specific cell collection and engineering [12,13].

Although CAR-based immunotherapy demonstrated substantial potential for cancer treatment, its initial clinical applications were accompanied by various adverse events. A lot of effort has been made in order to recognize the possible side effects and successfully manage them. The most common side effects include cytokine release syndrome (CRS), immune effector cell-associated neurotoxicity syndrome (ICANS), and cytopenias [14].

As CAR-based immunotherapies extend into new therapeutic areas and gain broader clinical application, the development of optimal strategies to manage associated side effects becomes increasingly important. One strategy for enhancing safety involves specifically targeting pathogenic cells while sparing non-pathogenic ones. This approach would prevent the mass depletion of an entire cell type or lineage and instead selectively target the true culprits. In line with this concept, antigen-based CARs and peptide-MHC (pMHC)-based CARs have been developed.

This review aims to examine the rationale, design, and therapeutic potential of advanced antigen-specific CARs in the context of hypersensitivity disorders, such as allergies and autoimmune diseases, highlighting their promise as safer and more precise immunotherapy options. Although allergies and autoimmune diseases differ in their clinical presentation, they share similar underlying immune dysregulations. Both conditions involve an aberrant immune response that targets either harmless environmental antigens or self-proteins, leading to chronic inflammation and tissue damage. This common immunological basis provides a strong rationale for the use of antigen-specific CAR therapies, such as pMHC-based CARs and CAARs which could offer targeted, precise, and safer treatment options for both types of hypersensitivity disorders.

## 2. Methods

### 2.1. Search Strategy

Although this review does not strictly follow the protocol of a systematic review, we conducted a rigorous and structured literature search to comprehensively analyze available studies on pMHC- and antigen-based CAR therapies for hypersensitivity disorders, including allergies, autoimmune diseases, and transplant rejection. In the first step, the review question was defined and translated into a systematic search strategy tailored to our research focus, as presented in Table 1. Subsequently, we conducted a structured electronic literature search. While our approach did not strictly adhere to the PICOTS framework, we applied a structured methodology to ensure a comprehensive and unbiased search. Specifically, we queried Medline (via Ovid), Embase, and Cochrane Central Register of Controlled Trials (CENTRAL) databases using predefined keyword combinations and Medical Subject Heading (MeSH)/Emtree terms, incorporating logical operators to identify relevant studies on CAR-based therapies for autoimmune diseases, hypersensitivity disorders, and transplant rejection. All databases were last searched on 3 February 2025. The complete search algorithm is provided in the Supplementary Materials (Table S1). The electronic database search yielded a total of 4615 potentially relevant studies for inclusion in this review, as illustrated in Supplementary Figure S1.

### 2.2. Screening and Data Extraction

Study screening and data extraction were performed by the first and last author (IPR and WFP) according to the inclusion and exclusion criteria. The inclusion and exclusion criteria, along with details on search terms and filters applied, are summarized in Table 2. Conflicts were resolved by discussion and achieving mutual consensus among the 2 reviewers. Data were extracted from each article by 1 of the 2 reviewers, with the other reviewer independently reading the article to ensure accuracy of data extraction. Variables used for data extraction were as follows: title, publication year, experimental setting, study design, primary and secondary aims of study, type of CAR construct, and targeted antigen. Quality assessment was performed by all authors to avoid a research bias in this literature review. Finally, of the 4615 studies identified through our structured search strategy, only 23 met the strict inclusion criteria relevant to CAAR and pMHC-based CAR therapies. Notably, none of these studies reported clinical trials involving these constructs in the context of autoimmune diseases, further underscoring the preclinical status of this highly specific therapeutic approach and supporting the reference selection presented in this review.

## 3. Targeting Specificity: CARs vs. CAARs vs. pMHC-Based CAR

This paragraph aims to provide an overview on the fundamental differences between classical CARs, nominal-antigen-based CARs, and peptide-MHC (pMHC)-based CARs. This section will explore their design principles and mechanisms of action while highlighting their unique advantages and limitations.

**Figure 1:**
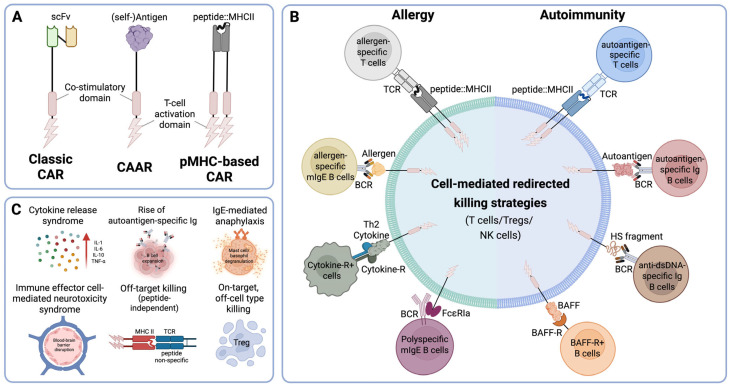
(**A**) Schematic structure of classical CARs, chimeric autoantibody receptor (CAAR), and peptide–MHC (pMHC)-based CARs. Classic CARs use a single-chain variable fragment (scFv) to recognize cell surface antigens. CAARs incorporate a (self-)antigen extracellular domain to selectively target autoreactive B cells. pMHC-based CARs present a peptide bound to MHC class II, enabling the targeting of antigen-specific CD4+ T cells via their TCR. (**B**) Overview of redirected cell-mediated killing strategies employed for redirecting cell-mediated cytotoxicity, including CAAR and pMHC-based CAR approaches, targeting autoreactive or allergen-specific T and B cells are illustrated. (**C**) Overview of possible toxicities associated with different CAR-based cellular immunotherapies.

CAR receptors are engineered to equip cytotoxic immune cells (αβ T cells, NK cells, or γδ T cells) with the ability to specifically recognize antigens expressed on the surface of target cells and eliminate them. The extracellular domain of the classical CAR recognizes targeted antigens, which are expressed multiple times on the surface of target cells, which leads to the cross-linking of the CAR with subsequent activation of the signal transduction modules represented by the intracellular domains (Figure 1A). Novel methods of recombinant DNA technology along with synthetic genomics opened new avenues to refine the construction of these sophisticated receptors in terms of their extra- and intracellular domains.

Nowadays, CAR signaling domains are often composed of separate activation and co-stimulatory domains, which have been borrowed from classical receptor molecules involved in sustained T-cell activation [15]. However, it began more simply with the use of the CD3ζ ITAM domain, marking the advent of the first-generation CARs [16], followed by the subsequent exploration of various chimeric constructs aimed at enhancing T-cell activation potential. One such study showed that the clustering of chimeric constructs containing the Syk kinase domain enhanced Ca^2+^ flux and cytolysis [17]. In contrast, chimeric constructs incorporating ZAP70 failed to induce similar effects unless co-clustered with Src kinases such as Lck or Fyn, which enabled them to overcome this limitation [17]. Therefore, designing constructs that incorporate active Src or Syk kinase domains along with critical phosphorylation sites from the ζ-chain or ZAP70 appeared advantageous. Such constructs can trans-phosphorylate each other upon antigen binding, and their dimerization could further enhance signaling, resulting in a more potent T-cell effector response [10]. While kinase-based signaling strategies improved initial activation, they lacked the ability to sustain long-term T-cell responses. To overcome this limitation, second-generation CARs incorporated co-stimulatory domains, such as CD28 or 4-1BB, which significantly enhanced signaling and efficacy [18]. Although cells expressing these CARs proved highly effective for treating B-cell leukemias and lymphomas, their efficacy in solid tumor therapy required further improvement. This led to the development of additional CAR generations, including the third generation that combined two co-stimulatory domains [19], the fourth-generation, i.e., TRUCK CARs, which combined direct antitumor action with the modulation of the tumor microenvironment through cytokine production [20], and the fifth-generation CARs, designed to enhance persistence and memory via JAK-STAT signaling pathways [21].

The extracellular CAR domain is responsible for targeting, endowing the modified cells with their intrinsic specificity. Traditionally, this domain is composed of variable heavy (VH) and variable light (VL) chains of monoclonal antibodies, connected by a flexible linker to form a single-chain variable fragment (scFv) most commonly linked via a (Gly_4_Ser)_3_ peptide [15,22]. These scFv-containing CARs enabled specific, high-affinity interactions of CARs with their antigens and MHC-independent activation of T cells [15] (Figure 1A). However, most of these scFv-based CARs were lineage-specific rather than selectively targeting pathogenic cells (CD20+ B cells, CD19+ B cells, BCMA^+^ B cells, CS1- SLAMF7+ plasma cells, CD5+ T cells, and CD7+ T cells) [23]. In recent years, significant efforts have focused on designing CARs that selectively target only pathogenic cells. One promising approach involves exploiting molecules that exhibit a natural affinity for one another, such as antigen–antibody interactions or peptide–MHC (pMHC) complexes interacting with T cells.

A pioneering study by T.L. Geiger et al. [24] explored a series of chimeric receptors containing the murine MHC class I molecule H-2 K^b^ linked to a variety of T-cell signaling domains that could specifically target the alloreactive T lymphocytes involved in graft rejection and graft-versus-host disease (Figure 1A,B). Peptides displayed in the context of the modified MHC molecules serve as bait, and together with the murine K^b^-molecule restricted their reactivity towards the TCRs of potentially pathogenic allo-antigen-specific T cells. The authors demonstrated that activation of signaling units in chimeric receptors was entirely dependent on their specificity for pathogenic TCRs. Notably, both coreceptor and costimulatory domains were shown to be crucial for effective signal transduction by these chimeric receptors [24].

Additionally, in their subsequent study [25], the same group demonstrated that antigen-specific K^b^ molecules linked to various signaling domains—TCR-ζ alone (K^b^-ζ), CD28 combined with ζ (K^b^-CD28-ζ), or CD28 and ζ along with Lck lacking its membrane attachment domain (K^b^-CD28-ζ-Lck)—could effectively redirect receptor-modified T cells (RMTCs) against antigen-specific T cells and induce their lysis. Notably, their findings demonstrated efficient antigen-specific cytolysis even at effector-to-target ratios below one, indicative of serial killing by CAR T cells [25]. Their research laid the foundation for incorporating natural molecular interactions into CAR engineering, highlighting the potential for highly specific and effective targeting of pathogenic cell populations.

To further understand the nature of RMTC, a study was conducted using receptor-modified T cells with the same signaling domains (CD28 and ζ) linked to either hen egg lysozyme (HEL) or the class I MHC K^b^ extracellular domains [26]. T cells equipped with either of these receptors showed the capacity to eliminate antigen-specific B cells both in vitro and in vivo. RMTCs effectively targeted cognate B cells via their B-cell receptor, even in the presence of saturating amounts of soluble immunoglobulins of the same specificity. Interestingly, although adoptive transfer of HEL-CD28-ζ T cells into wild-type mice effectively depleted antigen-specific B cells, it paradoxically also induced antibodies against the native form of the chimeric receptor antigen (i.e., HEL) [26]. These results demonstrated the broader applicability of the autoantigen-based CAR approach but also indicated its possible limitations. Different strategies have been employed to address this important issue, including the humanization of the CAR sequence, or the usage of natural receptor/antibody ligands [27]. This finding led to the development of the chimeric autoantibody receptor (CAAR) and opened the possibility of specifically targeting autoreactive B cells producing pathogenic autoantibodies.

Along these lines, one of the earliest successes was reported in 2016 by C. T. Ellebrecht et al. [28]. In their work, they designed human T cells that express a CAAR, consisting of the pemphigus vulgaris (PV) autoantigen, desmoglein (Dsg) 3, fused to CD137-CD3ζ signaling domains. Dsg3-containing CAAR demonstrated cytotoxicity against autoreactive B cells in vivo, even in the presence of circulating autoantibodies, without exhibiting off-target toxicity [28].

One potential advantage of pMHC-based and antigen-based approaches is their ability to target clonotypic receptors, such as disease-driving TCRs or BCRs, which are less likely to undergo antigen loss mutations. While pMHC-based CARs are restricted to specific HLA alleles, they do not necessarily depend on endogenous antigen processing and presentation pathways when the peptide is covalently linked to the MHC class II β-chain. This design ensures stable surface expression of the peptide–MHC complex independent of intracellular antigen processing, thereby enhancing targeting reliability [29,30,31]. Nevertheless, these limitations must be considered when designing novel CAR strategies for clinical application. Combining receptor-modified cells that target different pathogenic B or T cells could offer a robust strategy for eliminating autoreactive cells. This type of CAR design holds significant promise for the treatment of various hypersensitivity diseases, including allergies and autoimmune diseases. While the results to date are encouraging, specific safety concerns associated with these strategies must also be addressed when moving toward clinical translation. For CAAR T cells, the ectopic expression of membrane-bound autoantigens or allergens may pose a risk for further sensitization, leading to more instead of less auto- or allergen-specific antibody production, and thus fueling of the respective disease processes. In a similar vein, pMHC-based CARs, despite their targeting precision, may exhibit off-target activity by killing T cells in a peptide-independent manner or by inadvertently also depleting regulatory T cells (Tregs), potentially exacerbating immune dysregulation (Figure 1C). These considerations are critical when designing antigen-specific CAR therapies, and ongoing preclinical studies should continue to evaluate and mitigate these risks to ensure clinical safety. Table 3 provides an overview of relevant studies focused on the design and production of CAR cells based on the pMHC or CAAR principle, aimed at applications in allergy and autoimmune disease treatment, and also displays potential safety and toxicity concerns (see also Figure 1C).

## 4. Hypersensitivity Disorders

Hypersensitivity reactions are defined as immune responses that, although intended to protect the body, become exaggerated or inappropriate, leading to tissue damage [54]. Philip Gell and Robin Coombs proposed a classification for these reactions in 1963, which was later updated by Rajan Hopp [55]. The classification divides hypersensitivity reactions into four groups, as presented in Table 4.

### 4.1. Antigen-Based CARs in Allergy Treatment

The most common type of hypersensitivity is IgE-mediated allergy. This disease is caused by an exaggerated reaction of the immune system to different environmental antigens, mostly proteins, such as those present in pollen (tree, grass, weed), mold spores, cockroaches, animal dander, saliva, urine, insect venoms (bees, wasps, ants), foods (peanuts, tree nuts, milk, eggs, fish, shellfish, soy, wheat, fruits, vegetables), latex, and drugs [56]. Certain severe allergic disorders, such as asthma, atopic dermatitis, or multiple food allergies, significantly affect the quality of life of those impacted. Additionally, managing these diseases imposes substantial financial burdens on society. Allergic reactions represent hypersensitivity responses involving antibodies, immune cell-mediated mechanisms, as well as tissue-driven or metabolic processes, resulting in symptoms affecting the respiratory system, skin, eyes, gastrointestinal tract, and other organs. They may even lead to life-threatening anaphylaxis [56]. Upon initial sensitization by an allergen, allergen-specific IgE antibodies are produced. Subsequent exposures to the same allergen trigger noticeable allergic reactions once IgE levels reach a certain threshold. During the sensitization phase, antigen-presenting cells (APCs) capture and process allergens, subsequently presenting allergen-derived peptides in association with MHC molecules to naïve CD4^+^ T cells. APCs also secrete cytokines, such as IL-4, which drive the differentiation of naïve CD4^+^ T cells into the Th2 phenotype [56]. These Th2 cells subsequently secrete IL-4, IL-5, and IL-13. IL-4 and IL-13 promote immunoglobulin class switch recombination in B cells, leading to the production of allergen-specific IgE antibodies, and also enhance the migration of Th2 cells into tissues [56].

Allergen-specific immunotherapy (AIT) aims to induce (i) blocking IgG antibodies that neutralize allergens upon entry into the body and (ii) shift the pathological Th2-driven response against disease-causing allergens toward a protective Th1-type or regulatory (Treg)-mediated response. Currently, AIT remains the only curative treatment available for allergies. Over recent years, AIT has significantly advanced in terms of safety and patient convenience, while maintaining or even enhancing its therapeutic efficacy [57]. These innovations include among others the development of (i) hypoallergenic allergen variants and (ii) B-cell epitope vaccines, as well as (iii) optimization of adjuvants that favor protective immune responses. However, most AIT immunization schedules remain time-consuming, requiring substantial commitment and adherence from both patients and treating physicians due to multiple visits needed to achieve disease modification and clinical symptom improvement [58,59].

Consequently, other treatment options, primarily symptomatic, have been developed. One such treatment is based on Omalizumab, a monoclonal antibody that binds to the IgG-C-epsilon-3 domain, inhibiting IgE from binding to effector cells. This prevents effector cells from becoming activated, thereby reducing their responses upon subsequent allergen exposure.

However, due to its antibody-based nature, omalizumab must be administered repeatedly at intervals of 2–4 weeks, leading to considerable long-term treatment costs. Additionally, discontinuation of therapy results in an IgE rebound and recurrence of disease symptoms [60].

Indeed, targeting IgE and its associated signaling pathways could significantly advance the development of novel therapeutic approaches for allergy treatment, offering the potential to alleviate symptoms and improve patient quality of life [61].

Recently, a new approach aimed at eliminating the plasma cells responsible for IgE antibody production has been reported [62]. In their research using mouse models of allergy, the authors demonstrated that targeting long-lived plasma cells and preventing memory B cells from class-switching to IgE upon allergen exposure could represent a promising treatment approach for allergic diseases. They employed two monoclonal antibodies to target both existing and future IgE-producing B cells and plasma cells. Specifically, a bispecific CD3xBCMA antibody was used to temporarily eliminate all plasma cells and memory B cells, while the IL-4R-blocking antibody Dupilumab was employed to inhibit the activity of IL-4 and IL-13, the key cytokines involved in promoting IgE class-switching [62].

The above findings illustrate efforts to address the issue of exaggerated allergen-specific IgE production and/or enhanced immune activation of effector cells by targeting either the B-cell lineage or its IgE effector molecules. However, there is a scarcity of research strategies aimed at specifically engaging or targeting pathogenic allergen-specific T cells [10].

Employing adoptive cell transfer (ACT) therapies to specifically target IgE-producing B cells holds significant promise for achieving long-term relief from IgE-associated allergies [32]. These IgE-producing B cells can be targeted by engineered T cells recognizing the membrane-bound form of IgE, the IgE B-cell receptor (mIgE) [32]. One promising strategy involves the design of CARs incorporating the FcεRIα chain (FcεRIα-CAAR), specifically intended to bind mIgE [32]. FcεRIα was chosen due to its high-affinity interaction exclusively with the Cε3 domain of IgE antibodies (Kd ≈ 3.7 × 10^−10^ M) [32]. However, as reported by Ward et al., FcεRIα-based CARs expressed on T cells may become blocked by soluble IgE circulating in blood and tissues, preventing effective engagement with target mIgE^+^ B cells [32]. To overcome this limitation, the researchers designed CAARs incorporating FcεRIα mutants with reduced affinity for IgE [32]. Several point mutations were introduced into the FcεRIα domain, and the resulting novel receptor constructs were cloned into lentiviral vectors used to transduce Jurkat T cells [32]. Their study demonstrated that these low-affinity FcεRIα-mutant CAARs effectively bound to mIgE-expressing B cells but did not bind to soluble IgE antibodies [32]. Importantly, these mutant CAAR constructs also failed to recognize IgE bound to effector cells via FcεRI or FcεRII, thereby reducing the potential for severe off-target effects [32]. Nevertheless, a notable limitation of this FcεRIα-CAAR approach is its lack of specificity for allergen-specific mIgE-expressing B cells. Consequently, the authors acknowledged that using this type of CAAR might lead to the sustained suppression of all IgE-expressing B cells, potentially increasing patients’ susceptibility to parasitic infections [32].

Targeting IgE-producing B cells represents a promising strategy for mitigating allergic responses; however, recent advances in CAR-based approaches have broadened immunotherapy to include modulation of cytokine-producing cells involved in allergic inflammation. A groundbreaking study recently provided proof-of-concept and preclinical evidence demonstrating the potential for achieving long-term remission of asthma—a common chronic allergic disease—by employing engineered, long-lived, multifunctional T cells [33]. Asthma patients exhibit typical type 2 immune responses driven by IL-4-, IL-5-, and IL-13-producing T cells, which promote continuous IgE class switching in B cells, recruitment and differentiation of allergic effector cells such as eosinophils, and airway remodeling characterized by increased smooth muscle cells and enhanced mucus production [63]. These processes are mediated by infiltrating immune cells, including dendritic cells, eosinophils, neutrophils, lymphocytes, innate lymphoid cells (ILCs), and mast cells. Although IL-5 itself is not a chemoattractant, it mobilizes eosinophils from the bone marrow into peripheral tissues during allergic reactions [63].

Consequently, eosinophil depletion presents a promising therapeutic strategy for allergic asthma management. Engineered T cells expressing mouse IL-5-targeting CARs (5T cells) have been developed, with in vitro studies confirming their effective lysis of eosinophils and sustained eosinophil depletion [33]. These cells were enhanced through additional genetic modifications, including the simultaneous knockout of *Bcor* and *Zc3h12a* genes, enabling robust in vivo expansion (termed 5T_IF_ cells) and achieving prolonged eosinophil depletion, reduced lung inflammation, and amelioration of asthma symptoms for up to one year [33]. Furthermore, 5T_IF_ cells were modified with a mutated mouse IL-4 (mutein), generating 5T_IF_4 cells, capable of binding IL-4Rα without initiating downstream signaling, thereby inhibiting endogenous IL-4 and IL-13 signaling. [33]. Notably, cytokine release syndrome (CRS), a common adverse effect in human CAR therapies, was not observed in this mouse model [33]. However, increased monocyte and neutrophil levels were detected in the spleen one year after CAAR application, requiring future follow-up studies [33]. Interestingly, the abrogation of type 2 responses did not lead to clear shifts towards type 1 or type 3 immunity in this model [33].

These studies highlight the promising therapeutic potential of CAR-based immunotherapy for allergic diseases and underscore the importance of refining CAR designs to optimize specificity and safety, paving the way for improved therapeutic strategies.

As previously mentioned, Tregs are an important T cell subtype since they are the guardians of peripheral tolerance and have the ability to modulate undesired immune reactions under different circumstances, including autoimmunity, transplantation, and allergic diseases [64]. In fact, Treg-based therapies have been studied in autoimmune diseases in the past [65]. Combining CAR-based with Treg-based therapies could provide a potential solution for the treatment of IgE-mediated allergies [65]. This strategy involves engineering Tregs to express allergen-specific CARs targeting allergen-specific B-cell receptors [34]. M. Abdeladhim et al. designed Tregs that expressed ovalbumin (OVA) linked to CD28-CD3ζ (OVA-CAAR) to target B cells and sensitized mast cells [34]. The OVA-CAAR was tested in vivo in a murine OVA-alum allergy model, which results in measurable levels of anti-OVA IgE [34]. In their study, the authors also addressed questions regarding adverse reactions following treatment with the introduced OVA-CAAR. The study demonstrated that OVA-CAAR did not trigger anaphylactic reactions or excessive histamine release when administered to sensitized mice [34]. Furthermore, OVA-sensitized mice treated with OVA-CAAR were protected from anaphylaxis upon allergen challenge for at least 30 days [34]. Despite these promising results, the authors noted important limitations, including the inability of OVA-CAAR-Tregs to target resting B cells in bone marrow [34]. Additionally, they highlighted the challenge of protecting multisensitized individuals, as mast cells, basophils, and B cells can simultaneously bind IgE antibodies of multiple specificities via different Fcε receptors. Future studies are warranted to investigate potential bystander effects of CAAR Tregs in such complex clinical scenarios [34].

### 4.2. Antigen-Based CAR in the Treatment of Autoimmune Diseases

A similar necessity for specifically targeting disease-causing B or T cells exists for the treatment of severe autoimmunity. Autoimmune diseases (AIDs) represent a heterogeneous group of chronic diseases, which can manifest during childhood or at various stages of adulthood, and immune responses in these conditions may target individual organs or exert systemic effects [66,67]. The inability to induce self-tolerance in T or B cells promotes the development of AIDs [54,68]. Loss of tolerance occurs through the activation of T cells by antigen-presenting cells [69], which is either favored by molecular mimicry, peculiar cytokine milieus, or bystander activation during infectious diseases or chronic inflammation. After escaping central tolerance mechanisms in the thymus, they may migrate to the periphery and collaborate with self-reactive B cells. These B cells, through autoantigen-specific B-cell receptors, can capture, concentrate and present autoantigens to the autoantigen-specific T cells, leading to their activation [66]. While certain AIDs, like type 1 diabetes, primarily involve T cell-mediated tissue destruction, others cause significant harm though autoantibodies that induce complement activation and/or antibody-dependent cell-mediated cytotoxicity (ADCC) [66].

Currently, the management of AIDs relies largely on pharmacotherapy and monoclonal antibody-based biologics [70]. The growing demand for more precise targeting of autoreactive cells and increased therapeutic efficacy has recently shifted the research focus to CAR-based therapies. Indeed, broadly B-cell-targeting CAR strategies have shown initial promising outcomes in individual patient cases [71,72,73]. In addition to conventional CAR T cells, novel constructs are being explored to enhance safety and efficacy. For instance, RNA-engineered CAR T cells have demonstrated clinical activity in a phase 1b/2a study for myasthenia gravis without the need for lymphodepleting conditioning [74]. Allogeneic γδ T cell-derived CAR T products targeting the B-cell antigen CD20 are being tested in a phase 1 trial and may offer advantages due to their MHC-independence [75]. Furthermore, CAR-engineered regulatory T cells (CAR Tregs) targeting CD19 have shown the ability to restore immune homeostasis in preclinical models of systemic lupus erythematosus [76]. Moreover, currently, several early-phase clinical trials are underway to evaluate antigen-specific CAR T cell therapies in autoimmune diseases, such as in systemic lupus erythematosus, myasthenia gravis, and pemphigus vulgaris, marking a pivotal step towards the clinical translation of these promising approaches.

In light of these developments, it is essential to emphasize that the clinical application of CAR-based therapies for autoimmune diseases must be guided by structured evaluation and standardized protocols. Recently, expert-based recommendations for the implementation of innovative cellular therapies, including CAR T cell approaches, have been issued by the EBMT Practice Harmonization and Guidelines Committee [77]. These recommendations highlight the importance of multidisciplinary team evaluations involving both autoimmune disease specialists and cellular therapy experts to ensure appropriate patient selection and follow-up. They also provide detailed guidance regarding pre-therapy assessments, including general screening procedures, eligibility determination, and recommended drug-specific washout periods prior to leukapheresis and lymphodepletion. Furthermore, the document addresses strategies for supportive care and outlines the management of potential short-, medium-, and long-term complications, thereby supporting the safe and effective clinical implementation of CAR T-cell therapies.

However, despite these advances and the growing clinical interest, the development of therapies that specifically target autoreactive T lymphocytes remains a significant challenge. Antigen-specific approaches targeting limited antigenic epitopes often fall short of achieving the efficacy of antigen-nonspecific immunomodulatory or cell-eradicative strategies. Nevertheless, several strategies, including antigen-expressing CARs and peptide-MHC complex-expressing CARs, demonstrated therapeutic potential across different forms of AIDs. Table 3 provides an overview of the various CARs designed for AID therapy.

The first known chimeric receptor designed for the treatment of experimental allergic encephalomyelitis (EAE), a mouse model for multiple sclerosis, took advantage of the immunodominant myelin basic protein (MBP) peptide epitope, ranging from amino acids 89–101, which is restricted by Ia^s^ and was linked along with Ia^s^ to the cytoplasmic domain of the TCR-ζ chain [35]. The construct was expressed in transgenic mice under the control of the hCD2 promoter/locus-controlled region (LCR). The resulting CAR T cells, activated through their chimeric receptor by binding to antigen-specific T cells, effectively eliminated the latter in vitro. While CD8+ CAR T cells were therapeutically active, naive CD4+ cells were not. In fact, CD8+ pMHC-based CAR T cells proliferated upon encountering autoreactive T cells, inhibited their proliferation, and altered their cytokine secretion profiles. In vivo administration of these receptor modified T cells (RMTCs) significantly reduced EAE severity, lowered mortality rates, and induced sustained therapeutic responses, highlighting their substantial potential for EAE treatment.

Building on these findings, further investigations examined the role of CD4+ T cells differentiated into Th1 or Th2 phenotypes [36]. Research demonstrated that naïve CD4+ T cells equipped with pMHC-based CARs and differentiated towards a Th2 phenotype effectively alleviated EAE. IL-10 was identified as the key cytokine responsible for inhibiting autoreactive T-cell proliferation and disease activity. Furthermore, pMHC-based naïve CD4+ T cells induced autoreactive T cells to produce IL-4, subsequently promoting differentiation of naïve cells towards a therapeutic Th2 phenotype. This laid the foundation for the development of antigen-specific therapies based on the delivery of immunomodulatory/inhibitory IL-10 to pathogenic T cells.

Additionally, this research prompted studies exploring the therapeutic potential of CD4+CD25+ regulatory T cells (Tregs) modified with pMHC-based CARs in modulating autoreactive T cells within the EAE model [37]. These cells were effective in the treatment of EAE after its initiation through the adoptive transfer of pathogenic effector T cells. Moreover, the CARs demonstrated effectiveness even after epitope spreading, as evidenced by improved clinical scores, reduced mortality, and sustained remission lasting over 80 days post-treatment. The therapeutic potential of pMHC-modified CD4+CD25+ Tregs was further validated in vivo through the induction of IL-10-producing antigen-specific Tregs capable of suppressing autoimmunity independently of the original RMTCs [38]. The findings obtained in the EAE model underscore the potential of modifying distinct T-cell populations with CARs, opening new avenues for targeted therapeutic interventions in autoimmune diseases.

A significant challenge in the development of antigen-specific therapies for autoimmunity was the comparatively lower affinity of self-reactive TCRs relative to foreign antigen-reactive TCRs [39], likely due to selection pressures in thymic negative selection and regulatory T-cell mechanisms. A study employing myelin oligodendrocyte glycoprotein (MOG)_35–55_ pMHCII-CARs primarily reacting with high-affinity TCRs successfully prevented the onset of EAE but failed to achieve the elimination of low-affinity autoreactive T-cell clones; thus, the reversal of established disease was not achieved. By increasing pMHCII stability and introducing modifications that improved CAR T-cell survival, the study authors were able to target both high- and low-affinity autoreactive T cells, significantly reducing EAE severity. These results support a model in which T cells with high-affinity TCRs act as initiators of autoimmunity, while lower-affinity T-cell clones maintain chronic inflammation [39]. pMHC-based CAR strategies thus present a promising approach not only for EAE but also for other autoimmune conditions such as rheumatoid arthritis [50].

In addition to the pMHC CAR-based approach, CAAR-based approaches have also shown promise in the treatment of AIDs. As previously mentioned, this approach was used for the first time in 2016 for the treatment of pemphigus vulgaris [28]. Since then, this therapeutic principle has been applied to other autoimmune disorders, including MuSK-positive myasthenia gravis [49], primary biliary cholangitis [51], bleeding disorders such as hemophilia A [52], and primary immune thrombocytopenia [53]. In addition, the CAAR-based approach was explored as a potential therapy for EAE, where MBP was fused extracellularly to CD137 and CD3ζ signaling domains [40]. These MBP CAAR T cells demonstrated in vitro cytotoxic effects even at low effector-to-target ratios. Furthermore, IFN-γ, IL-2, and TNF-α cytokines were only detected when MBP CAAR T cells were co-cultured with target cells, suggesting that they are produced as a response to the formation of immunological synapses between MBP CAAR T and autoreactive B cells [40].

Another AID that affects the central nervous system (CNS) is encephalitis, which is caused by autoantibodies against the N-methyl-D-aspartate (NMDA) receptor (NMDAR). The modulation of NMDR by these autoantibodies results in signaling deficiencies, severe disturbance of autonomous brain functions, and psychiatric symptoms. NMDAR autoantibodies are formed by short-lived plasmablasts that are constantly replenished by differentiating memory B cells [78]. In order to eliminate such autoantibody-producing B cells/plasmablasts, a CAAR was developed by carefully selecting several epitopes from the NMDAR to ensure broad targeting of pathogenic B cells [41]. T cells equipped with this extracellular domain, which consisted of several subunits and was linked to the intracellular 4-1BB/CD3ζ domains, had been shown to be effective in reducing autoantibody levels in serum and cerebrospinal fluid in a passive transfer model in vivo without showing signs of off-target toxicity or adverse events. A key advantage of this approach over monoclonal antibody therapies is the suggested ability of CAAR T cells to penetrate the CNS, as has been demonstrated for lymphoma-targeting CARs [79]. This highlights the advantage of cell-based therapies in achieving deeper tissue penetration, even in areas to which anti-B-cell monoclonal antibodies such as rituximab have limited access. They may also minimize the side effects of chronic immunosuppression by focusing exclusively on the elimination of pathogenic B cells.

One of the most relevant and studied AIDs is type 1 diabetes mellitus (T1D). Approximately 74 million adults worldwide and around 300,000 children in the WHO European Region are affected by T1D [80]. Furthermore, WHO estimates suggest that one in ten individuals in the region will develop diabetes by 2045 [80]. The first autoantigen that was found in patients with T1D was insulin itself [81]. It has been shown that the destruction of insulin-producing β cells in the pancreas is driven by aberrant CD8+ T cells that recognize immunodominant insulin epitopes [82]. Targeting these pathogenic T cells using CAR-based therapies presents a promising long-term treatment strategy with the potential to halt disease progression in affected individuals. Just this was attempted when a CAR was designed using the immunodominant insulin peptide ranging from amino acids 15–23 linked to β2-microglobulin and the CD3ζ chain as the intracellular signaling domain. This construct was then expressed in mouse CD8+ T cells (InsCD3ζ T cells) [42]. These pMHC-based CARs effectively eliminated diabetogenic insulin-reactive CD8+ T cells. In vitro experiments revealed that cytotoxicity was mediated by both perforin- and FAS-FAS ligand-mediated apoptosis. Notably, InsCD3ζ T cells successfully targeted insulin-specific CD8+ T cells in an early stage of disease development, thereby reducing if not preventing disease progression. Furthermore, InsCD3ζ T cells did not influence other autoantigen-specific T cells, such as islet-specific glucose-6-phosphatase catalytic subunit-related protein (IGRP)-reactive CD8+ T cells. These findings highlight the need for developing CARs targeting multiple autoantigens to allow for the effective treatment of T1D.

Building on the promising results of targeting autoantigen-specific T cells, further advancements have been made in optimizing CAR T-cell therapies. One such innovation is the application of electroporation of mRNA to introduce genes of interest into primary cells, which is a rapid, simple, and highly efficient method, ensuring high and uniform gene expression under mild, non-cytotoxic conditions [43]. This method not only preserves cell viability but also enables the simultaneous introduction of multiple genes as pre-defined combinations into T cells [43]. It has been used to express multiple specific peptide/β2 microglobulin/CD3ζ CARs in reporter B3Z T cells [43]. Beyond insulin (immunodominant Insulin_15–23_ peptide), other important autoantigens include islet-specific glucose-6-phosphatase catalytic subunit related protein (IGRP_206–214_ dominant peptide), dystrophia myotonica kinase (DMK_138–146_ peptide), and glutamic acid decarboxylase (GAD_546–554_ peptide). In addition to the effective transfection of B3Z cells, the mRNA method was also used to successfully transfect primary mouse T cells with multispecific CAR vectors. After confirming the in vitro cytotoxicity of peptide/β2-microglobulin/CD3ζ T cells, in vivo studies were conducted. Notably, a reduction in diabetes incidence in mice treated with Ins_15–23_/β2 microglobulin/CD3ζ T cells was observed; however, neither Ins_15–23_/β2 microglobulin/CD3ζ nor IGRP_206–214_/β2 microglobulin/CD3ζ T cells showed the reducing effect. These results suggest that CAR T cells with different targeting specificities may need to be administered at different stages of disease development to achieve optimal therapeutic outcomes [43].

Recent studies have also shown that Treg cells expressing CAR effectively redirect autoreactive CD8+ T cells in the T1D mouse model [45]. CD4+ Treg cells were reprogrammed by electroporation of mRNA constructs encoding peptide/β2-macroglobulin/CD3-ζ, incorporating either a modified InsB_15–23_ or IGRP_206–214_ peptide. The generated cells expressed FoxP3 along with other typical Treg lineage markers and secreted IL-10. Driven by autoantigen recognition, peptide-MHC CAR Treg cells migrated to pancreatic lymph nodes and Langerhans islets. In vitro experiments revealed their ability to suppress CD8+ T-cell proliferation, induce activation and exhaustion, upregulate regulatory markers on CD8+ T cells, and promote apoptosis in a subset of CD8+ T cells. In vivo, these cells provided protection against diabetes and mitigated insulitis severity in a T1D mouse model. Since this strategy targets cells in the early stage of the disease, careful consideration is required for its translation to human applications [45].

Another promising strategy for enhancing CAR efficacy and specificity involves the use of biomimetics to develop chimeric receptors that replicate the natural operating mechanisms of T cells. This innovative chimeric module (CRM) consists of an extracellular peptide-MHCII domain (module 1) and the intracellular domains of the TCRα and TCRβ subunits, allowing integration with the natural CD3γε, δε, and ζζ modules, which form the sheath of each TCR (modules 2–4) [44]. In addition, a surrogate coreceptor was added as a fifth module, comprising the CD80 extracellular domain linked via a transmembrane domain to p56^Lck^ (Lck). This module was incorporated to enhance signaling strength through CD80–CD28 or CD80–CTLA-4 interactions [44]. Both in vitro and in vivo studies have shown that this novel CRM effectively eliminates CD4+ autoreactive T cells in T1D mice, with cytotoxicity driven by proliferative expansion of CAR T cells and IFN-γ dependent, TCR-specific killing. The study emphasized that multiple refinements will be necessary to optimize CRM technology. Further comparative analyses with other CAR strategies and a deeper understanding of T-cell activation mechanisms will be essential for the development of next-generation biomimetic CARs.

Systemic lupus erythematosus (SLE) is among the most complex autoimmune diseases in terms of clinical presentation, genetic predisposition, and immunological mechanisms [83]. It is a condition marked by episodic flare-ups of pain and inflammation, affecting multiple organ systems [84]. At the molecular level, SLE is characterized by dysregulated B-cell activation, autoantibody production, and nephritis [46,85]. The disease is driven by the hyperactivation of autoreactive B cells, resulting in the production of autoantibodies targeting double-stranded (ds) DNA and nuclear protein autoantigens. These antibodies, produced by plasmablasts and long-lived plasma cells, form immune complexes that cause tissue damage and organ dysfunction upon deposition [83]. B-cell development is a step-wise process governed by distinct immunoglobulin gene rearrangement processes [86]. In addition, growth and differentiation factors control the development of B cells, with B-cell activating factor (BAFF), a TNF superfamily cytokine [46], playing a particularly important role. BAFF receptor (BAFFR) expression begins as immature B cells transition into mature B cells, where BAFFR-mediated survival signaling is essential for preventing premature apoptosis [86]. In SLE, overproduction of BAFF leads to increased activation and survival of B cells, which contributes to the expansion of autoreactive B-cell clones that are more prone to autoantibody production [87]. Recent advances in CAAR T immunotherapy have shown promise in enabling targeted immunomodulation that could lead to remission in SLE. One particularly promising approach involves the generation and development of BAFF CAAR T cells [46]. These BAFF-based CAAR T cells were engineered using a truncated BAFF sequence encompassing the majority of the extracellular domain of the natural human BAFF ligand, fused to intracellular CD28, OX40/CD134 costimulatory domains, the CD3ζ signaling domain, and the CD28 transmembrane domain [88]. A construct lacking BAFF but identical in all other aspects was designed to serve as a negative control (no-BAFF control). The BAFF CAAR construct was expressed in primary mouse CD8+ T via lentivirus transduction [46]. After demonstrating the in vitro cytotoxicity of BAFF CAAR T cells against B cells isolated from SLE patients, including CD19+ IgM+ cells, the authors assessed their therapeutic efficacy in vivo using three distinct SLE mouse models: the genetically predisposed spontaneous MRL/lpr model, pristane-induced SLE in BALB/c mice, and an SLE patient-derived xenograft (PDX) model, which involves injecting peripheral blood mononuclear cells (PBMCs) into immunocompromised NOD SCID gamma (NSG) mice [46]. Mice treated with BAFF CAR T cells showed persistent depletion of mature B cells, with minimal effects on precursor B cells. This resulted in a significant reduction in autoantibody production and an improved clinical course of SLE compared to mice treated with no-BAFF control T cells. In particular, BAFF CAR T cells effectively killed human CD19+ B cells in the humanized SLE PDX model.

Despite the potential therapeutic benefits of BAFF CAAR T cells in SLE treatment, there are significant concerns regarding long-term humoral immunity. Due to their ability to target mature B cells, memory B cells, and antibody-producing plasmablasts, BAFF CAAR T cells could cause prolonged immunosuppression, leading to severe consequences for patients. As the authors noted, potential side effects need to be carefully investigated in future clinical trials to ensure for safety and efficacy of the therapy [46].

To better address the problem of specificity and immunosuppression during SLE treatment, DNA CAAR T cells have been developed [47]. These DNA CAAR T cells express antigens that are designed to eliminate anti-dsDNA-producing B cells. Several kidney tissue antigens, including α-actinin, heparan sulfate, histone-1, and C1q, are known to exhibit high cross-reactivity with dsDNA, potentially contributing to the autoimmune response in SLE [47]. Accordingly, six chimeric autoantibody receptors (DNA-CAAR) with autoantigen protein sequences of α-actinin (*n* = 2, DNA1 or DNA2 CAAR), heparan sulphate (*n* = 2, DNA3 or DNA4 CAAR), histone-1 (*n* = 1, DNA5 CAAR), and C1q protein (*n* = 1, DNA6 CAAR) fused to the activator domains CD28–CD3ζ were developed and expressed in primary human T cells [47]. Due to low expression levels of DNA5, only the cytotoxic effect of DNA CAARs 1–4 and 6 could be investigated in vitro against B cells from patients with anti-dsDNA^+^ antibodies. Notably, DNA4 and DNA6 CAAR T cells exhibited the highest levels of specific cytotoxicity. Experiments using kidney organoids revealed significant morphological improvements following treatment with DNA4 CAAR T cells, including reduced organoid cell death and decreased expression of renal damage markers such as kidney injury molecule-1 (KIM-1). Confocal imaging further demonstrated that DNA4 CAAR T cells established substantial cell-to-cell contact with pathological B cells within two hours, whereas DNA6 CAAR T cells required six hours of co-incubation to achieve similar interactions. These promising findings suggest that DNA CAAR T cells may play a crucial role in advancing future lupus nephritis treatments.

Another renal AID, i.e., membranous nephropathy (MN), is characterized by the presence of autoantibodies targeting phospholipase A2 receptor 1 (PLA2R1) and thrombospondin type-1 domain-containing protein 7A (THSD7A) [48]. To selectively eliminate autoantibody-producing cells, CAAR NK-92 cells expressing the immunodominant regions of MN antigens PLA2R1 and THSD7A were developed [48]. These newly designed CAAR NK-92 cells exhibited a dose-dependent cytotoxic effect against pathogenic B cells and maintained their cytotoxic potential even in the presence of anti-PLA2R1 and anti-THSD7A monoclonal antibodies. However, further in vivo studies are required to adequately determine their therapeutic potential [48].

## 5. Concluding Remarks: Challenges and Future Strategies

In summary, pMHC-based and antigen-based CAR T-cell therapies represent promising remedies for the treatment of autoimmune diseases and allergies. These therapies offer the potential for precise targeting of antigen-specific B or T cells within the body, allowing for personalized treatment approaches and potentially reducing the adverse effects commonly associated with conventional therapies. Although these treatment modalities are still in the research phase, they have demonstrated efficacy in preclinical models in vivo and have shown the potential to revolutionize current treatment strategies, providing patients with improved control over immune responses. However, further advancements in this therapy are clearly necessary. For instance, the field of allergy treatment is desperately awaiting pMHC-based allergen-specific approaches to efficiently target allergen-specific but sparing non-allergen specific T cells. In principle, the knowledge about immunodominant allergen peptides of major allergens for the development of such CARs and the human-relevant preclinical models for the evaluation of their efficacy is readily available. Now is the right time for proof of principle studies along those lines. Another necessity regards the improvement of the safety, efficiency, and the accessibility of CAR-based ATMPs to a wider population. Additionally, the advent of in vivo gene delivery technologies for generating CAR, CAAR, and pMHC-based CAR T cells holds great promise and is currently being explored in early-phase clinical studies. This breakthrough has the potential to revolutionize the field by enabling more efficient and targeted therapeutic interventions for autoimmune diseases. Nevertheless, continued research and a multi-disciplinary approach to the development of these therapies will be crucial in realizing their full potential in the treatment of allergies and autoimmune diseases.

## Figures and Tables

**Table 1:** Key questions guiding the literature review.

Category	Review Question
Targeting Conditions	What hypersensitivity reactions (allergies, autoimmune diseases) are being addressed using pMHC CAR or antigen-based CAAR therapies?
CAR Design	What are the key characteristics of pMHC CAR and CAAR constructs designed for these indications?
Preclinical and Clinical Development	What stages of development (preclinical models, clinical trials) exist for these therapies?
Efficacy and Safety	What are the reported benefits, challenges, and safety concerns of pMHC CAR and CAAR therapies for treating hypersensitivity-related diseases?

**Table 2:** Inclusion and exclusion criteria for data screening and extraction.

Category	Criteria
Inclusion criteria	Studies investigating pMHC CAR or CAAR-based therapies Preclinical (in vitro, in vivo) and clinical studies. Studies focusing on autoimmune diseases, hypersensitivity disorders, or transplant rejection. Articles published in peer-reviewed journals. English-language publications
Exclusion criteria	Studies using scFv-based CARs Studies not related to antigen-specific or HLA or MHC-specific CAR therapy Reviews, commentaries, editorials, or conference abstracts Non-English publications
Databases searched	Medline (via Ovid), Embase, Cochrane CENTRAL
Last search date	3 February 2025

**Table 3:** An overview of different CAR designs based on pMHC or CAAR platforms for the treatment of allergy or autoimmunity.

Disease	CAR Type (Extracellular Domain)	Signaling Domains	Targeted Cells	Experiment Setting	Total Doses Administered *	Achieved Result *	Safety Considerations	Citation
Allergy	CAAR-Jurkat T cells (human FcεRIα, and its 5 mutants)	CD3ζ	Cells expressing membrane-bound IgE (mIgE)	In vitro	-	-	n.a.	[32]
	CAAR-CD8+ T cells (mouse IL-5 and mouse IL-4 mutein)	CD28-CD3ζ	IL-5Rα^+^ cells	In vitro In vivo (mouse model)	Single intravenous injection	Long-term remission of asthma	No CRS or toxicity reported	[33]
	CAAR-Tregs (ovalbumin)	CD28-CD3ζ	anti-OVA IgE-producing B cells	In vivo (mouse model)	Single intravenous injection	Protection from hypothermia and anaphylaxis	No acute toxicity or anaphylaxis reported	[34]
Experimental allergic encephalomyelitis (mouse model of multiple sclerosis)	pMHC-based CAR- CD8+ T cells (Immunodominant peptide epitope of the myelin basic protein (MBP) autoantigen class II MHC IAs chain)	CD3ζ	Autoreactive T cells	In vitro In vivo (mouse model)	Single dose	Elimination of autoreactive T cells	No safety data reported	[35]
	pMHC-based CAR- CD4+ T cells (MBP autoantigen class II MHC IAs chain)	CD3ζ	Autoreactive T cells	In vitro In vivo (mouse model)	Single intravenous injection	Effective in treating advanced EAE, IL-10 is crucial for the therapeutic activity	No safety data reported	[36]
	pMHC-based CAR- CD4+ CD25+ regulatory T cells (MBP autoantigen class II MHC IAs chain)	CD3ζ	Autoreactive T cells	In vitro In vivo (mouse model)	Single intravenous injection	Suppression of EAE	No safety data reported	[37]
	pMHC-based CAR- CD4+ CD25+ regulatory T cells (MBP autoantigen class II MHC IAs chain)	CD3ζ	Autoreactive T cells	In vitro In vivo (mouse model)	Single intravenous injection	Suppression of the autoreactive T cell response and EAE development in an IL-10-dependent manner	No safety data reported	[38]
	pMHC-based CAR CD8+ T cells (MOG_97–108_ pMHCII (DR4)-CAR)	CD28-CD3ζ	Auto antigen-specific CD4+ T cells	In vitro In vivo (mouse model)	Single intravenous injection	Naïve and activated CD4+ T cells can be efficiently eliminated in vivo using pMHCII-CAR cells in a TCR-specific manner	No safety data reported	[39]
	CAAR (myelin basic protein (MBP)	CD137-CD3ζ	Autoreactive B cells	In vitro	-	-	n.a.	[40]
NMDAR encephalitis	CAAR Jurkat T cells/primary human T cells (NMDAR autoantigen)	4-1BB-CD3ζ	Autoreactive B cells	In vivo in vivo (mouse model)	Single dose	A reduction in serum and brain autoantibodies with no signs of off-target toxicity or adverse events	No off-target toxicity reported	[41]
Diabetes type 1	pMHC-based CAR CD8+ T cells (insulin peptide 15–21 linked to β2-microglobulin)	CD3ζ	Autoreactive T cells	In vitro In vivo (mouse model)	Single intravenous injection	Reduced insulitis and diabetes after adoptive transfer of insulin-reactive CD8 T cells into NOD.SCID mice	No safety data reported	[42]
	pMHC-based CAR mouse CD8+ T cells (peptide/β2-microglobulin/peptide: Ins_15–23_, IGRP_206–214_)	CD3ζ	T cells expressing insulin-reactive G9C8 or IGRP-reactive NY8.3 TCRs	In vitro In vivo (mouse model)	Single intravenous injection	Protection when InsB_15–23_/β2m/CD3-ζ mRNA-transfected cells were transferred targeting InsB_15–23_-reactive T cells	No safety data reported	[43]
	pMHCII-based chimeric module receptor CTLs (pMHCII and CD80 extracellular domains; Peptide: InsB_9–23_; HIP2, HIP6)	TCRα and TCRβ subunits assembled with the CD3γε, δε, and ζζ linked to the pMHCII extracellular domain; p56^Lck^ (Lck) linked to CD80 extracellular domain;	Autoreactive T cells	In vitro In vivo (mouse model)	Single dose	Effective in eliminating CD4+ autoreactive T cells in T1D mouse model with cytotoxicity driven by IFN-γ production, proliferation, and cell killing in a TCR-specific manner	No signs of toxicity or pathology in mice reported	[44]
	pMHC-based CAR CD8+ T cells (peptide/β2-microglobulin peptide: Ins_15–23_, IGRP_206–214_)	CD3ζ	Autoreactive T cells	In vitro In vivo (mouse model)	Two doses one-week interval	Protection against diabetes and reduced the severity of insulitis	No safety data reported	[45]
Systemic lupus erythematosus **	CAAR CD8+ T cells (B-cell activating factor (BAFF))	CD28-OX40-CD3ζ	Autoreactive B cells	In vitro In vivo (mouse model)	Single dose	Complete lysis of SLE patient plasma B cells in mouse model	No safety data reported	[46]
Lupus nephritis	DNA CAART (α–actinin, heparan sulphate, histone-1, and C1q)	CD28-CD3ζ	B cells expressing anti-dsDNA autoantibodies	In vitro	-	-	n.a.	[47]
Membranous nephropathy	CAAR-NK-92/T cells (Immunodominant regions of the MN antigens PLA2R1 and THSD7A)	membrane-proximal ITAM domains of human CD28, the ITAM domain of human 4-1BB, and the CD3ζ	Autoreactive B cells	In vitro	-	-	n.a.	[48]
Pemphigus Vulgaris **	CAAR T cells (desmoglein (Dsg) 3), or combination Dsg3 with extracellular cadherin (EC) domains1-3, 1-4, and 1-5)	CD137-CD3ζ	Autoreactive B cells	In vitro In vivo (mouse model)	Single dose	Decreased Dsg3 serum autoantibody titers, absence of autoantibody binding and blistering in oral mucosa	No off-target toxicity reported	[28]
Muscle-specific tyrosine kinase myasthenia gravis **	CAAR T cells (Muscle-specific tyrosine kinase (MuSK))	CD137-CD3ζ	Autoreactive B cells	In vitro In vivo (mouse model)	Single dose	Reduced anti-MuSK IgG without decreasing B cells or total IgG levels, reflecting MuSK-specific B cell depletion	No off-target toxicity reported	[49]
Rheumatoid arthritis	pMHC-based CAR CD8+ T cells (peptide from type II collagen (CII) linked to the DRB1 chain, linked to second domains from I-E^d^	CD28-CD3ζ	Autoreactive T cells	In vitro In vivo (mouse model)	Two doses	Autoimmune CD4+ T cell response decreased, autoantibody production suppressed, and the incidence and severity of autoimmune arthritis diminished	No safety data reported	[50]
Primary biliary cholangitis	CAAR CD8+ T cells (PD-L1)	CD28-CD3ζ	PD-1+ T cells	In vitro In vivo (mouse model)	Two doses	Depleted liver CD8+ T cells and alleviated autoimmune cholangitis	No off-target toxicity; no pathological changes in multiple organs; minimal impact on systemic inflammation reported	[51]
Hemophilia A	CAAR Tregs (Human FVIII C2 or human A2)	CD28-CD3ζ	FVIII-specific B cells	In vitro	-	-	n.a.	[52]
Primary immune thrombocytopenia	CAAR Jurkat (GPIba)	4-1BB-CD3ζ	Autoreactive B cells	In vitro In vivo (mouse model)	Single dose	Selectively lysed target cells, led to reduced autoantibody titers, and a lower human platelet clearance rate in a xenograft mouse model.	No off-target toxicity reported	[53]

* These data apply exclusively to in vivo experiments. ** These are Phase 1 clinical trials currently in the recruitment stage, identified via ClinicalTrials.gov. n.a., not applicable.

**Table 4:** Classification of hypersensitivity reactions and their corresponding diseases.

Hypersensitivity Type	Characteristic	Disease
Type 1 (IgE mediated)	Immediate Orchestrated by Th2 cells Local or systemic	Asthma Allergic rhinitis Atopic eczema Urticaria Food Allergy Anaphylaxis
Type 2 (Antibody-mediated)	IgM and IgG antibodies bind to cell surface molecules, resulting in activation of the complement system or antibody-dependent cell-mediated cytotoxicity	Autoimmune hemolytic anemia Immune thrombocytopenia Hemolytic disease of the newborn Rheumatoid fever Good posture syndrome Rheumatic fever Pernicious anemia Pemphigus Myasthenia gravis Graves’ disease
Type 3 (Immune complex-mediated)	Formation of antibody complexes that bind to basal membranes Systemic	Systemic lupus erythematosus Post-streptococcal glomerulonephritis Polyarthritis nodosa Serum sickness
Type 4 (Cell-mediated)	Delayed Activation of T cells by tissue antigen-presenting cells (APCs) leading to tissue damage	Contact dermatitis Hashimoto thyroiditis Multiple sclerosis (MS) Rheumatoid arthritis (RA) Type1 diabetes Guillain–Barre syndrome Celiac disease Crohn’s disease Graft-versus-host disease (GVHD) Autoimmune myocarditis

## Data Availability

No new data were created.

## Supplementary Materials

The following supporting information can be downloaded at https://www.mdpi.com/article/10.3390/cells14100753/s1: Table S1: Literature search algorithm (Medline via Ovid); Figure S1: Flow diagram depicting the literature screening approach.
