# Supplementary material for: Chimeric Autoantibody Receptor- and/or Peptide-MHC-Based CAR Therapies for Targeted Elimination of Antigen-Specific B or T Cells in Hypersensitivity Disorders Such as Allergies and Autoimmune Diseases
Source: Cells. 2025 May 21;14(10):753. doi: 10.3390/cells14100753 (PMC12110022; doi:10.3390/cells14100753)
Supplement: Supplementary file 1 [file cells-14-00753-s001.zip › Figure S1_IPR_2025_02_28.pdf]

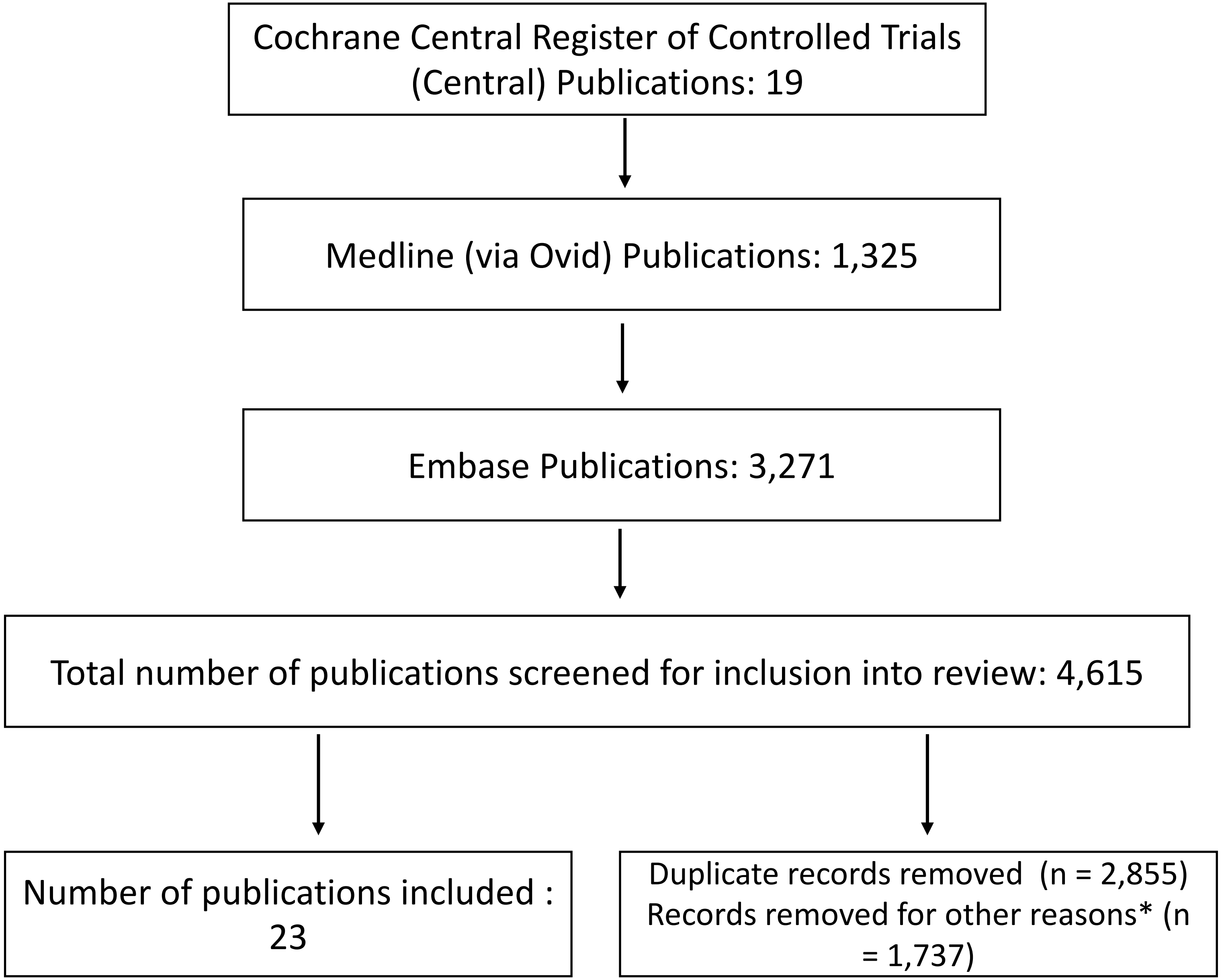

*Creteria for publication removal: Studies using scFv-based CARs, studies not related to antigen-specific or HLA or MHC-specific CAR therapy, reviews, commentaries, editorials, or conference abstracts, non-English publications

Figure S1: Flow diagram depicting the literature screening approach
