# Supplementary material for: Chimeric Autoantibody Receptor- and/or Peptide-MHC-Based CAR Therapies for Targeted Elimination of Antigen-Specific B or T Cells in Hypersensitivity Disorders Such as Allergies and Autoimmune Diseases
Source: Cells. 2025 May 21;14(10):753. doi: 10.3390/cells14100753 (PMC12110022; doi:10.3390/cells14100753)
Supplement: Supplementary file 1 [file cells-14-00753-s001.zip › Table S1_IPR_2025_02_28.pdf]

Table S1. Literature search algorithm (Medline via Ovid)

#	Query
1	("antigen specific*" or "allergen specific*" or "hla specific*" or "autoantigen specific*") and (car or cars or "chimeric antigen receptor*" or "chimeric receptor*").ti,ab,kf.
2	((pmhc or peptideMHC or "peptide MHC" or "peptide major histocompatibility complex" or pmhcII or "peptideMCHII" or "peptide MHCII") and (car or cars or "chimeric antigen receptor*")).ti,ab,kf.
3	(caar* or "chimeric autoantibody receptor*" or "chimeric hla antibody receptor*").ti,ab,kf.
4	((car or cars or "chimeric antigen receptor*") adj3 therap*).ti,ab,kf.
5	("receptor modified t cell*" or RMTC).ti,ab,kf.
6	"adoptive cell transfer*".ti,ab,kf.
7	("long lived car" or "long lived cars").ti,ab,kf.
8	"adoptive t cell therap*".ti,ab,kf.
9	"gene modified t cell*".ti,ab,kf.
10	"transgenic t cell*".ti,ab,kf.
11	1 or 2 or 3 or 4 or 5 or 6 or 7 or 8 or 9 or 10
12	exp autoimmune diseases/
13	exp hypersensitivity/
14	exp Graft vs Host Disease/
15	(autoimmun* or "auto immun*").ti,ab,kf.
16	("Systemic Lupus Erythematosus" or "Lupus Erythematosus Disseminatus" or "Libman Sacks Disease").ti,ab,kf.
17	("multiple scleros*" or "Disseminated Scleros*").ti,ab,kf.
18	(("type 1" or "type one" or "type I" or "insulin dependent") adj1 diabet*).ti,ab,kf.
19	"Rheumatoid Arthriti*".ti,ab,kf.
20	thyroidit*.ti,ab,kf.

21	("graft vs host" or "graft versus host").ti,ab,kf.
22	(reject* adj4 transplant*).ti,ab,kf.
23	(allerg* or hypersensitiv*).ti,ab,kf.
24	12 or 13 or 14 or 15 or 16 or 17 or 18 or 19 or 20 or 21 or 22 or 23
25	11 and 24
26	limit 25 to english
